# Molecular Characterization and Expression Analyses of the Complement Component *C8*α, *C8*β and *C9* Genes in Yellow Catfish (*Pelteobagrus* *fulvidraco*) after the *Aeromonas* *hydrophila* Challenge

**DOI:** 10.3390/ijms17030345

**Published:** 2016-03-08

**Authors:** Huan Zheng, Wei Ji, Gui-Rong Zhang, Xiao-Ting Zhang, Ze-Chao Shi, Kai-Jian Wei, Rui-Bin Yang, Jonathan P. A. Gardner

**Affiliations:** 1 Key Laboratory of Freshwater Animal Breeding, Ministry of Agriculture, College of Fisheries, Huazhong Agricultural University, Wuhan 430070, China; zhenghyt@126.com (H.Z.); wei-ji@mail.hzau.edu.cn (W.J.); grzhang@mail.hzau.edu.cn (G.-R.Z.); zxt1003@126.com (X.-T.Z.); rbyang@mail.hzau.edu.cn (R.-B.Y.); Jonathan.Gardner@vuw.ac.nz (J.P.A.G.); 2 Freshwater Aquaculture Collaborative Innovation Center of Hubei Province, Wuhan 430070, China; shizechao@yfi.ac.cn; 3 Key Laboratory of Freshwater Biodiversity Conservation, Ministry of Agriculture, Yangtze River Fisheries Research Institute, Chinese Academy of Fishery Sciences, Wuhan 430070, China; 4 School of Biological Sciences, Victoria University of Wellington, P O Box 600, Wellington 6140, New Zealand

**Keywords:** *Pelteobagrusfulvidraco*, complement component, *C8α*, *C8β*, *C9*, cDNA cloning, gene expression, bacterial challenge

## Abstract

The complement components C8α, C8β and C9 have important roles in the innate immune system against invading microorganisms. Partial cDNA sequences of the *Pf_C8α*, *Pf_C8β* and *Pf_C9* genes (*Pf*: abbreviation of *Pelteobagrus* *fulvidraco*) were cloned from yellow catfish. The *Pf_C8α*, *Pf_C8β* and *Pf_C9* genes showed the greatest amino acid similarity to C8α (54%) and C8β (62%) of zebrafish and to C9 (52%) of grass carp, respectively. Ontogenetic expression analyses using real-time quantitative PCR suggested that the three genes may play crucial roles during embryonic and early larval development. The mRNA expressions of the three genes were all at the highest levels in liver tissue, and at lower or much lower levels in 16 other tissues, demonstrating that the liver is the primary site for the protein synthesis of *Pf_*C8α, *Pf_*C8β and *Pf_*C9. Injection of *Aeromonas* *hydrophila* led to up-regulation of the three genes in the spleen, head kidney, kidney, liver and blood tissues, indicating that the three genes may contribute to the host’s defense against invading pathogenic microbes. An increased understanding of the functions of the *Pf_C8α*, *Pf_C8β* and *Pf_C9* genes in the innate immunity of yellow catfish will help enhance production of this valuable freshwater species.

## 1. Introduction

The complement system, which is made up of a series of distinct serum proteins and cell surface receptors [1], is an essential link between innate and adaptive immune responses that allows the host defense against bacterial invasion and inflammatory response [2,3,4]. There are three pathways initiated by different triggers to activate the complement system: the classical, the alternative and the lectin [5]. All three pathways lead to the formation of the membrane attack complex (MAC), which is made up of the terminal complement component (TCC) plasma proteins C5b, C6, C7, C8, and multiple C9 [6]. The MAC, formed on cell membranes, is effective in causing lysis of a variety of target cells through a multi-hit process in innate immune response [2].

The C5b-C9 TCCs are a group of structurally related plasma proteins, but varied somewhat in complexity and size. They belong to the membrane attack complex and perforin (MACPF) super family [7]. Specifically, the TCCs have some common structural motifs, including the thrombospondin domain (TSP), the low-density lipoprotein receptor class A domain (LDLR-A), the unique membrane attack complex/perforin-like domain (MACPF) and the epidermal growth factor-like domain (EGF) [8].

The complement C8 has three genetically distinct subunits, including C8 alpha (C8α), C8 beta (C8β) and C8 gamma (C8γ) [9], which are encoded by three different genes [10]. C8α and C8β belong to the same family and have close homology to the C6, C7 and C9 proteins. C8γ belongs to the lipocalin family, but it is not essential for the MAC-mediated cytolytic activity [11]. C8α is required for lysis because it provides a binding site for C9 [11]. It traverses the lipid bilayer as the first protein, leading to the formation of membrane-penetrating “pores” [12]. C8β is required for cell lysis, as it mediates C8 to bind to C5b-7, a precursor of the MAC [13]. C8β deficiency is strongly associated with neisserial infections, and may lead to deficiency of the complement cytolytic activities [14]. Therefore, C8β plays important roles in the immune defense system [15]. In addition, C9 is known to be the major component for the formation of a pore-like structure that is characteristic of the fully formed MAC [16].

The innate immune system is a fundamental defense system in fish and the only defense system in invertebrates. It is more critical for immunity in teleosts than in mammals [17]. Accumulating evidence indicates that a primitive complement system is identified in cnidarians [18], arthropods [19], mollusks [20], sea urchins [21] and ascidians [22], whereas cartilaginous and bony fishes possess a modern complement system [23]. In teleosts, several genes in the lytic pathway have been cloned and characterized at molecular levels. The complement *C9*, *C7*, *C8α* and *C8β* in rock bream (*Oplegnathus* *fasciatus*), and *C9* and *C7* in grass carp (*Ctenopharyngodon* *idella*) were identified and characterized at the genomic level [24,25,26,27,28]. In zebrafish (*Danio rerio*), the differential infected *versus* non-infected expression of *C6*, *C8α* and *C9* transcripts in fins, head kidney, spleen and liver were studied shortly after infection-by-immersion with viral hemorrhagic septicemia virus (VHSV) [29]. In addition, the complement *C9*, *C6*, *C7*, *C8α* and *C8β* genes in rainbow trout (*Oncorhynchus mykiss*) [30,31,32,33,34], *C6* gene in grass carp [35], and *C8α*, *C8β* and *C9* genes in carp (*Cyprinus* *carpio*) [36,37] have been cloned and characterized. These studies indicate that TCC genes are essential for the fish immune defense system. Recent studies have shown that the complement activation pathways in bony fishes are fully developed, and that they play crucial roles in innate immunity [38].

Yellow catfish (*Pelteobagrus* *fulvidraco* Richardson) (Teleostei: Bagridae) is an important commercial fish in the aquaculture industry of China due to its excellent meat quality [39]. In recent years, artificial aquaculture of yellow catfish has been seriously affected by several kinds of bacterial diseases, including ascites disease [40], ulcerative syndrome [41], and red-head disease [42], all of which have led to substantial economic losses. In the innate immune response, complement-mediated killing of invading pathogenic microbes through the lytic pathway is one of the major effector mechanisms [27]. The final MAC assembly disrupts the target cell by forming a membrane-penetrating “pore” in the lipid bilayer [43,44]. Therefore, it is extremely important to understand the expression behavior of TCC genes when exploiting the fish immunity system to prevent diseases. In this study, sequences of the *Pf_C8α*, *Pf_C8β*, and *Pf_C9* genes (*Pf*: abbreviation of *Pelteobagrus* *fulvidraco*) from yellow catfish were identified using rapid amplification of cDNA ends (RACE) based on transcriptome data. We investigated the tissue distributions of *Pf_C8α*, *Pf_C8β* and *Pf_C9* genes in adults and their expression profiles during the early larval developmental stages using real-time quantitative PCR (qPCR). Furthermore, tissue-specific expressions were carried out to determine the responses of *Pf_C8α*, *Pf_C8β* and *Pf_C9* genes to challenges of *Aeromonas* *hydrophila*.

## 2. Results

### 2.1. Characterization of Pf_C8α, Pf_C8β and Pf_C9 cDNA Sequences

**Figure 1:**
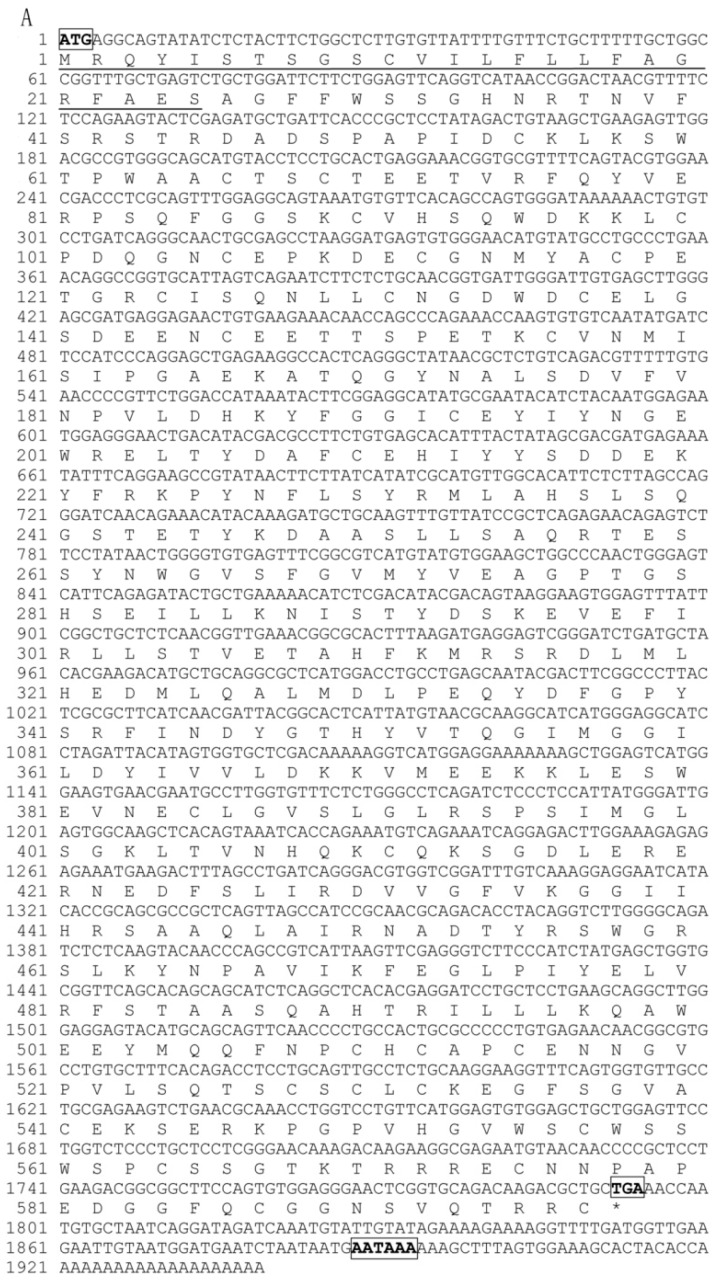

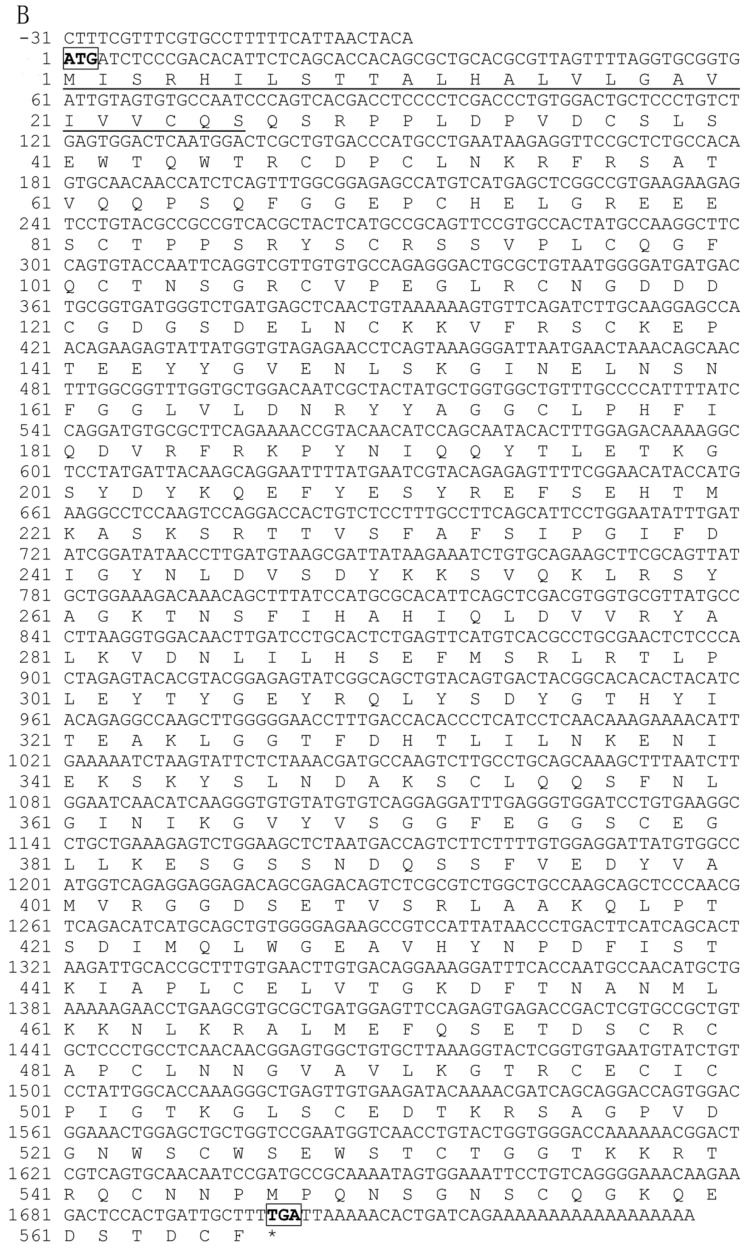

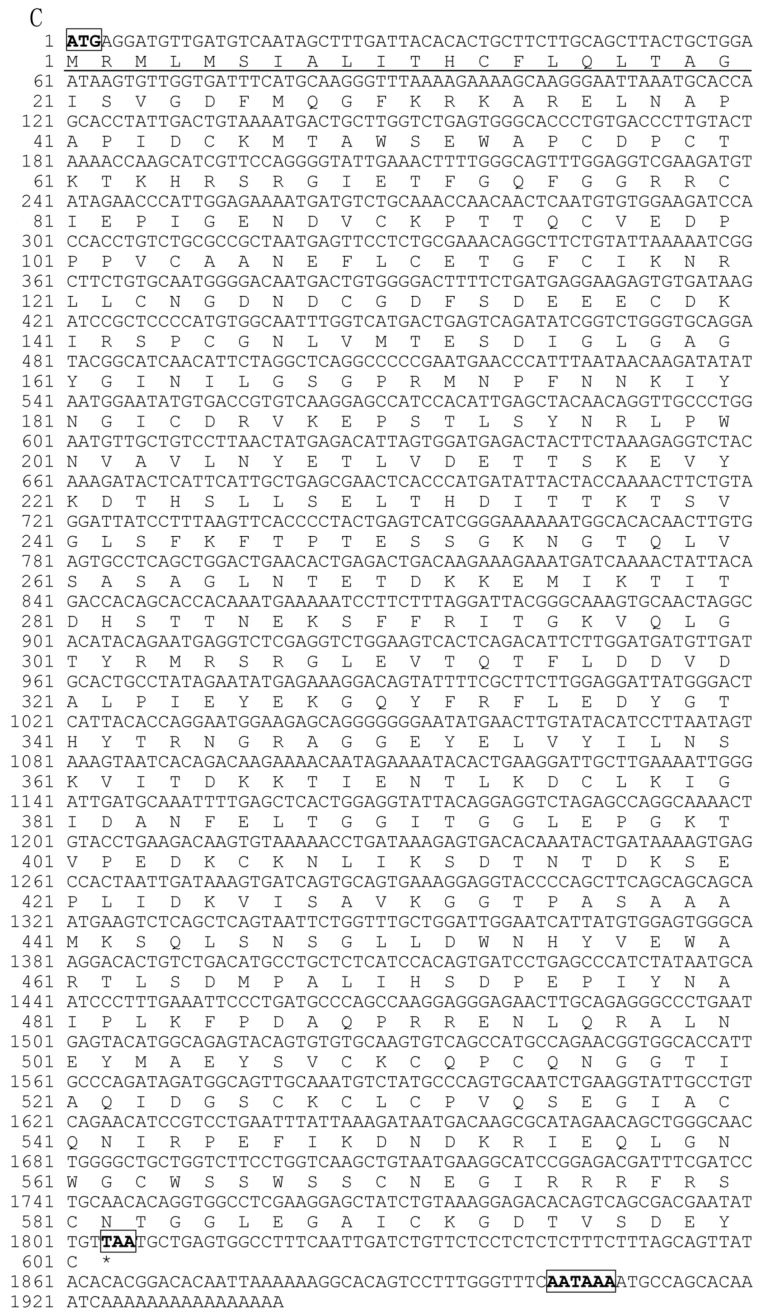
Partial nucleotide and deduced amino acid sequences of *Pf_C8α* (**A**); *Pf_C8β* (**B**) and *Pf_C9* (**C**). The nucleotide sequence (**upper** line) and the deduced amino acid sequence (**lower** line) are numbered. Start codons, stop codons and polyadenylation signal sequences (AATAAA) are indicated by boxes. Predicted signal peptides are underlined. * indicates stop codon.

The partial cDNA sequences of *Pf_C8α*, *Pf_C8β* and *Pf_C9* genes were cloned from yellow catfish by RACE-PCR (Figure 1). The partial cDNA of the *Pf_C8α* gene (GenBank accession no. KT588317) was 1939 bp, and contains a 1794 bp open reading frame (ORF) and a 145 bp 3′-untranslated region (UTR), with a 20 bp poly (A) tail and a polyadenylation signal (AATAAA) (Figure 1A). The ORF of the *Pf_C8α* gene encoded 597 amino acids, of which the first 25 residues were predicted to be a signal peptide. The molecular mass and isoelectric point (pI) of the amino acids were estimated to be 67.25 kDa and 5.858, respectively.

The partial cDNA of the *Pf_C8β* gene (GenBank accession no. KT588318) was 1767 bp, and contains a 31 bp 5′-UTR, a 1701 bp ORF, and a 35 bp 3′-UTR, with an 18 bp poly (A) tail (Figure 1B). The ORF of the *Pf_C8β* gene encoded 566 amino acids, of which the first 26 residues were predicted to be a signal peptide. The estimated molecular mass and pI of the amino acids were 63.24 kDa and 6.534, respectively.

The partial cDNA of the *Pf_C9* gene (GenBank accession no. KT454382) was 1939 bp, and contains an 1806 bp ORF and a 133 bp 3′-UTR, with a 16 bp poly (A) tail and a polyadenylation signal (AATAAA) (Figure 1C). The ORF of the *Pf_C9* gene encoded 601 amino acids, of which the first 20 residues were predicted to be a signal peptide. The estimated molecular mass and pI of the amino acids were 66.59 kDa and 5.478, respectively.

### 2.2. Homology Comparison and Phylogenetic Analyses of Pf_C8α, Pf_C8β and Pf_C9

**Figure 2:**
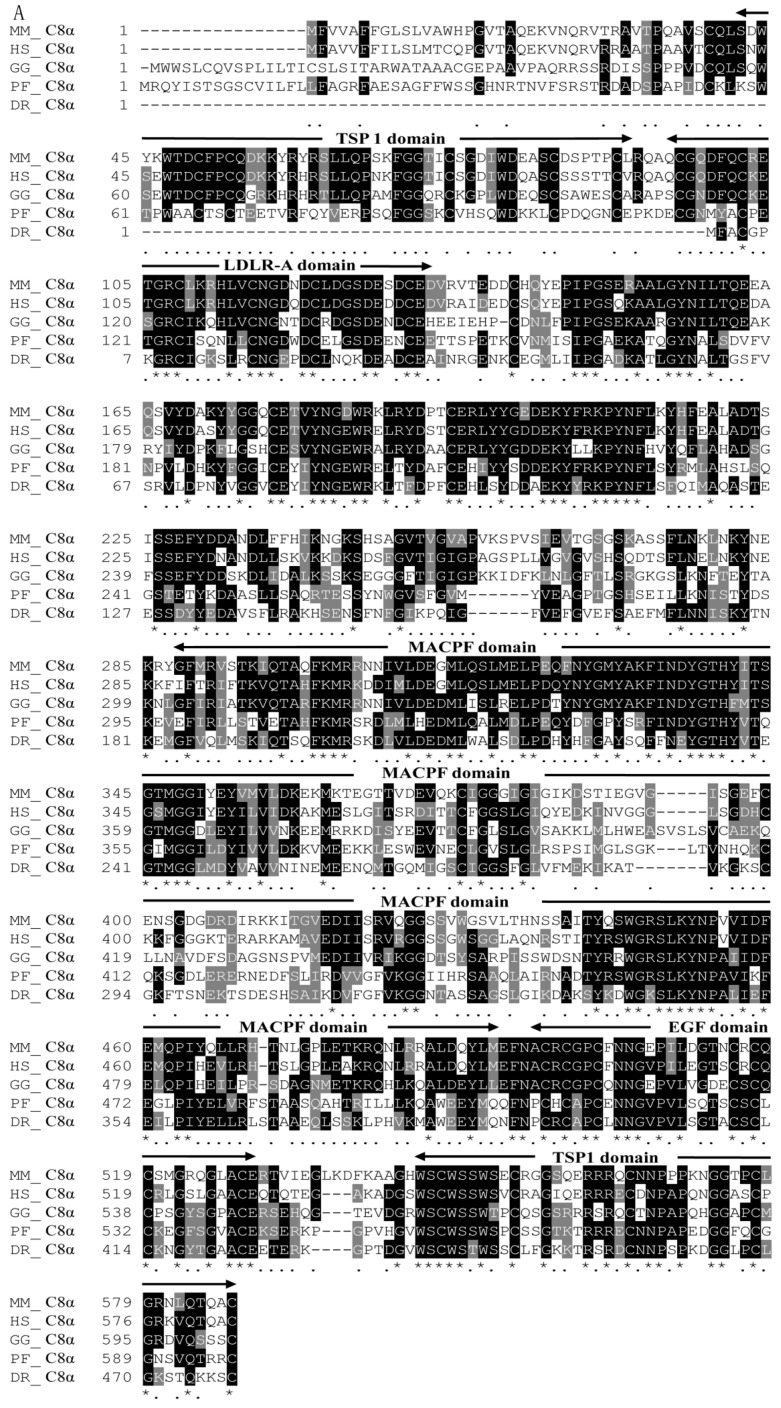

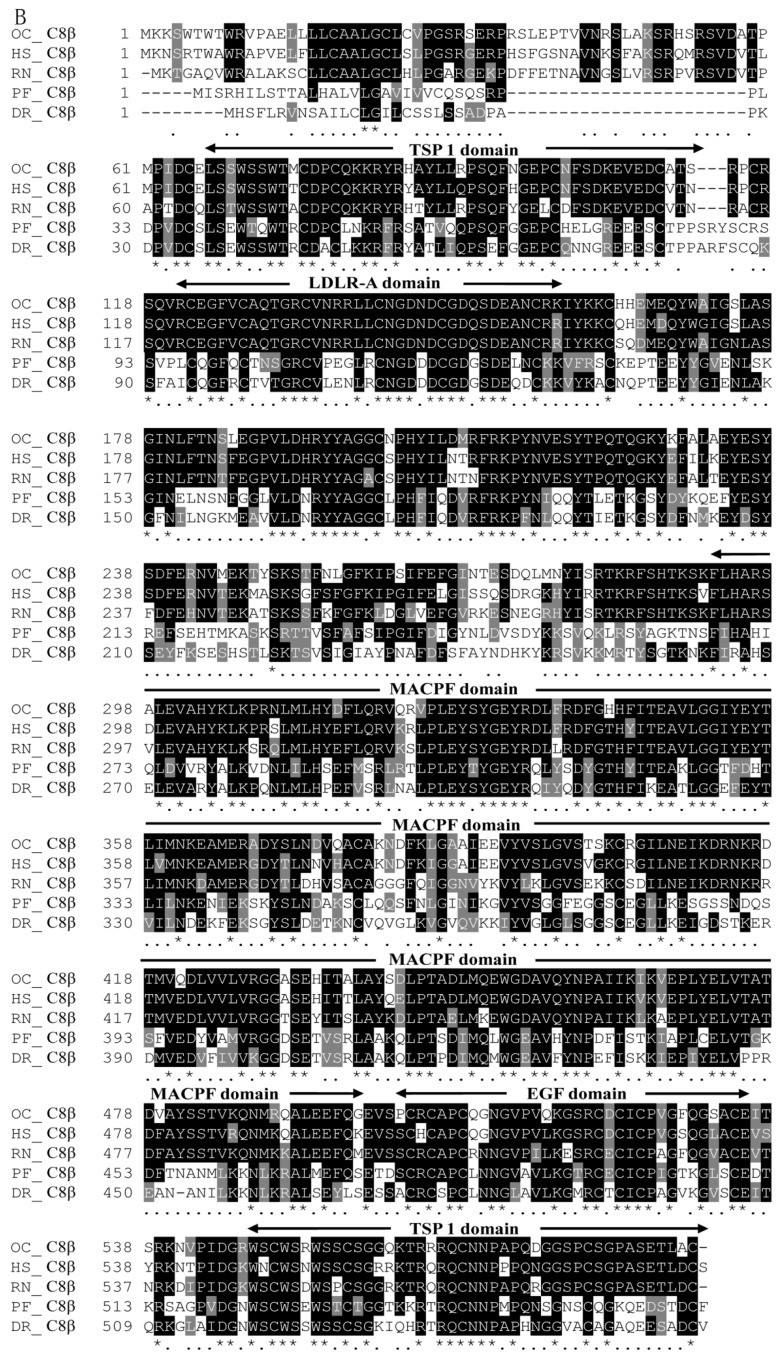

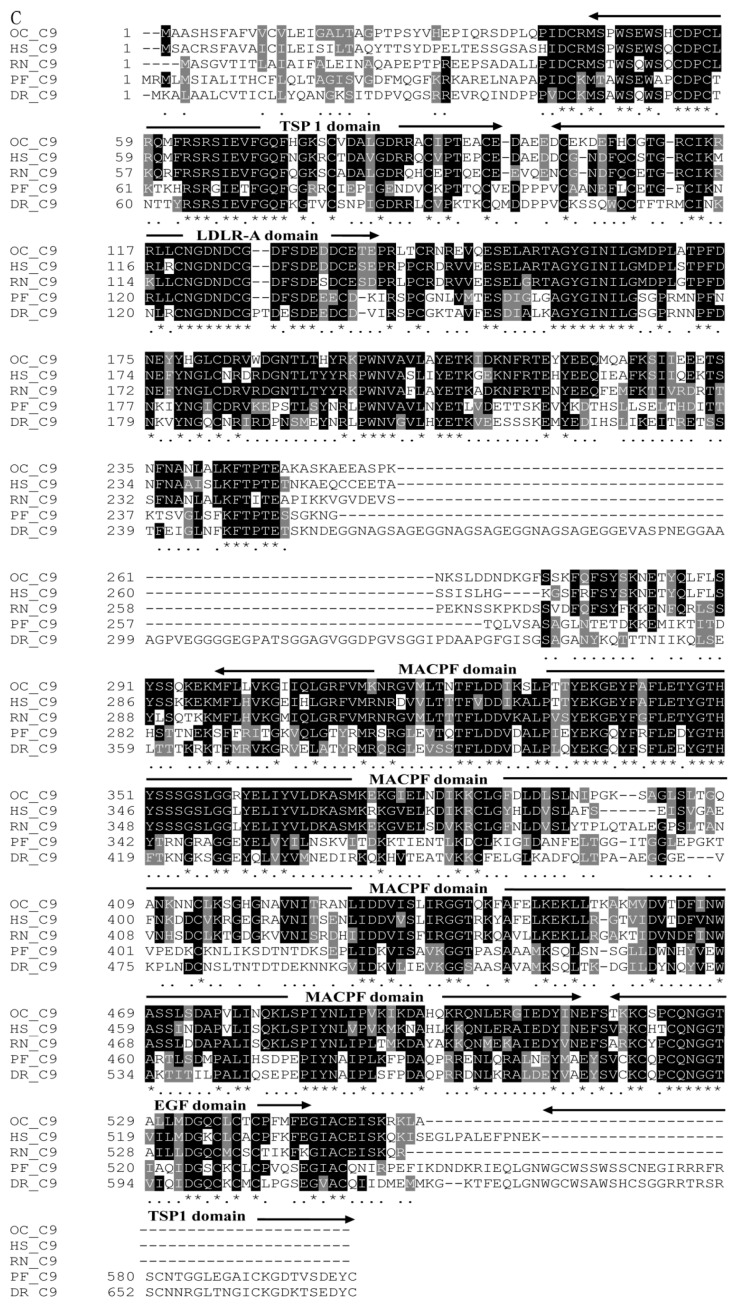
Multiple sequence alignment of the deduced amino acids of *Pf*_C8α (**A**); *Pf_*C8β (**B**) and *Pf_*C9 (**C**) with other vertebrates, derived using the ClustalW program. The identical residues are indicated in black and marked with an asterisk (*). Conserved and semi-conserved residues are indicated by gray shading, marked with a semicolon (:) and a dot (.), respectively. Missing amino acid is marked by dashes; the conserved domain is indicated by double arrows. MM_C8α: *Mus musculus* C8α; HS_C8α: *Homo sapiens* C8α; GG_C8α: *Gallus gallus* C8α; PF_C8α: *Pelteobagrus* *fulvidraco* C8α; DR_C8α: *Danio rerio* C8α; OC_C8β: *Oryctolagus* *cuniculus* C8β; HS_C8β: *Homo sapiens* C8β; RN_C8β: *Rattus* *norvegicus* C8β; PF_C8β: *Pelteobagrus* *fulvidraco* C8β; DR_C8β: *Danio rerio* C8β; OC_C9: *Oryctolagus* *cuniculus* C9; HS_C9: *Homo sapiens* C9; RN_C9: *Rattus* *norvegicus* C9; PF_C9: *Pelteobagrus* *fulvidraco* C9; DR_C8β: *Danio rerio* C9. The GenBank accession numbers of these sequences are shown in Table 1. TSP: thrombospondin; LDLR-A: low-density lipoprotein receptor class A; MACPF: unique membrane attack complex/perforin-like; EGF: epidermal growth factor-like.

BLAST (Basic Local Alignment Search Tool) analysis in GenBank showed that the *Pf_*C8α amino acid sequence had the highest homology (54%) to that of *D. rerio* (Accession No. AAH78409.1), followed by *Siniperca* *chuatsi* (Accession No. AKA66305.1) (52%) and *O. mykiss* (Accession No. NP_001118096.1) (51%). Motif Scan analysis identified four potential functional domains in the *Pf_*C8α amino acid sequence: TSP 1 (57–107 amino acids and 555–597 amino acids), LDLR-A (111–148 amino acids), MACPF (298–505 amino acids) and EGF (509–542 amino acids) (Figure 2A).

BLAST analysis revealed that the *Pf_*C8β amino acid sequence had the highest homology (62%) to that of *D. rerio* (Accession No. NP_001243652.1), followed by *Esox* *lucius* (Accession No. XP_010885201.1) (61%) and *O. mykiss* (Accession No. AAL16647.1) (60%). The Motif Scan analysis revealed four potential functional domains in the *Pf_*C8β amino acid sequence of yellow catfish: TSP 1 (39–86 amino acids and 523–566 amino acids), LDLR-A (96–132 amino acids), MACPF (266–473 amino acids) and EGF (477–510 amino acids) (Figure 2B).

BLAST analysis showed that the *Pf_*C9 amino acid sequence had the highest homology (52%) to that of *C. idella* (Accession No. ABN49522.1), followed by *O. mykiss* (Accession No. NP_001117898.1) (50%) and *O. fasciatus* (Accession AFU81223.1) (49%). The motif scan analysis identified four potential functional domains in the *Pf_*C9 amino acid sequence: TSP 1 (47–97 amino acids and 561–597 amino acids), LDLR-A (103–140 amino acids), MACPF (289–504 amino acids) and EGF (508–541 amino acids) (Figure 2C).

**Figure 3:**
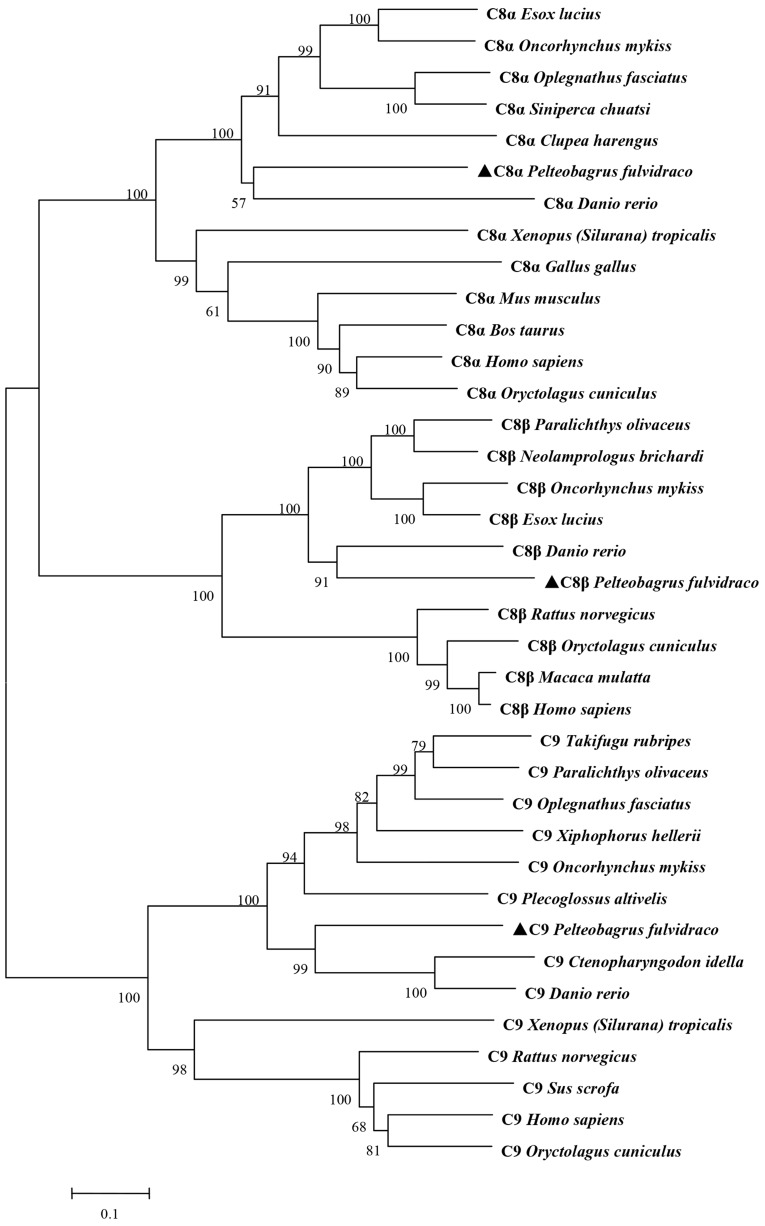
Neighbor-joining (NJ) phylogenetic relationship of *Pf*_C8α, *Pf_*C8β and *Pf_*C9 with those of other vertebrates. The phylogenetic tree was constructed based on the deduced partial amino acid sequences of *Pf_*C8α, *Pf_*C8β and *Pf_*C9 using the neighbor-joining method in MEGA 5.03. The numbers at tree nodes indicate percentage of 1000 bootstrap samples. The scale bar refers to a phylogenetic distance of 0.1 amino acid substitutions per site. The GenBank accession numbers of these sequences are shown in Table 1.

To understand the evolutionary relationship of the *Pf_C8α*, *Pf_C8β*, *Pf_C9* genes with those of other vertebrates, a neighbor-joining (NJ) phylogenetic tree (Figure 3) was constructed based on deduced amino acid sequences. C8α, C8β and C9 clustered into two branches, one branch including teleost species and the other branch including mammalians and other vertebrates, respectively. The yellow catfish shared the greatest homology of C8α and C8β with zebrafish. *Pf_*C9 was clustered with those of zebrafish and grass carp, and they shared high homology (Figure 3).

### 2.3. Quantitative Analyses of Pf_C8α, Pf_C8β and Pf_C9 mRNA Expression Levels in Adult Tissues

**Figure 4:**
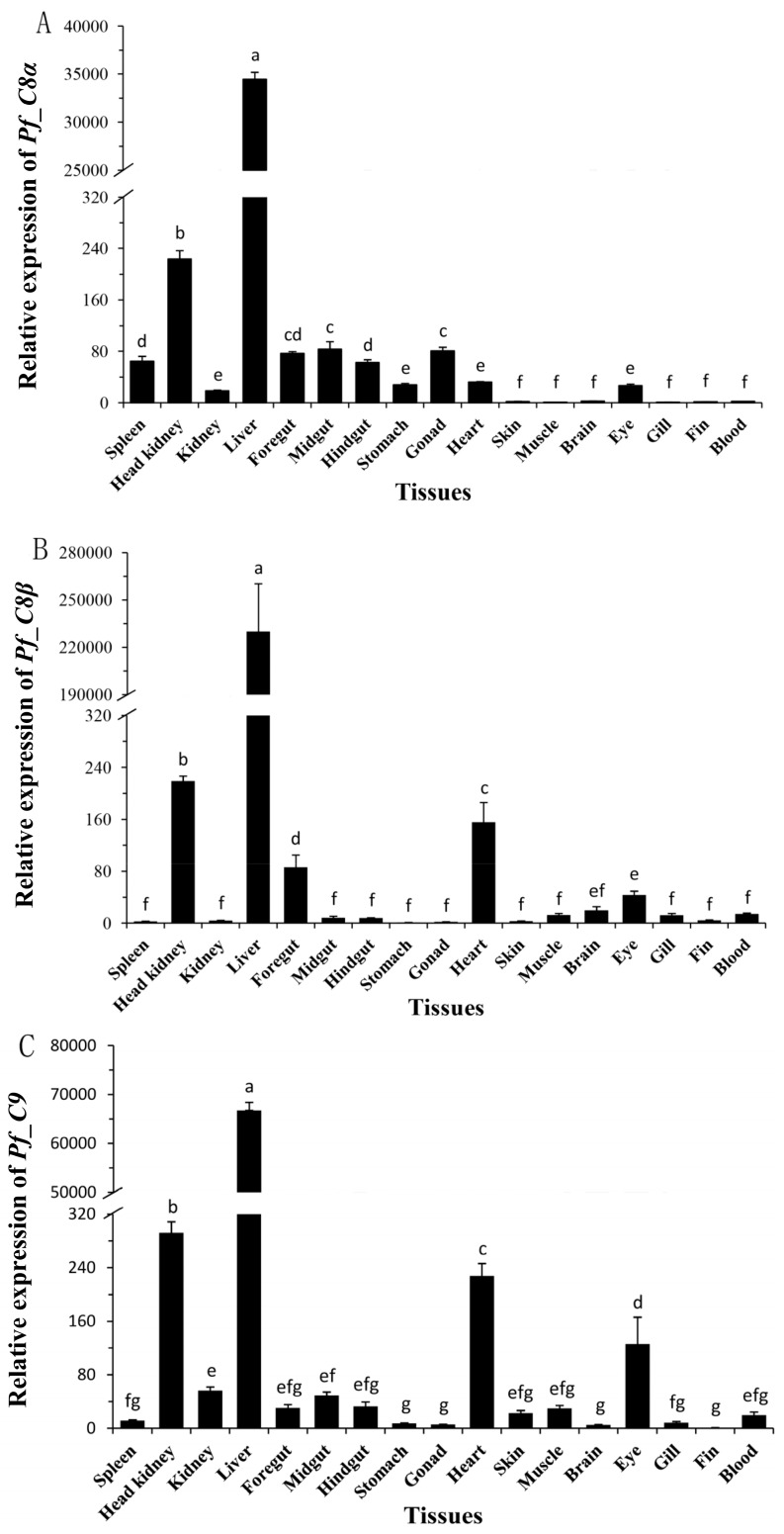
Tissue distributions of relative expression levels of *Pf_C8α* (**A**); *Pf_C8β* (**B**) and *Pf_C9* (**C**) mRNAs. Columns represent the means of 3 repeats for each treatment. Error bars represent standard error of the means. Different letters above the bars indicate significant difference (Duncan test, *p* < 0.05).

The tissue distributions of *Pf_C8α*, *Pf_C8β* and *Pf_C9* mRNA expressions were detected by qPCR, and calibrated by *β-actin* mRNA expressions in the corresponding tissues, including blood, liver, spleen, brain, head kidney, kidney, foregut, midgut, hindgut, stomach, heart, gill, muscle, skin, fin, eye, and gonad. Expression levels of all three genes were greatest in the liver, followed by a relatively low level in the head kidney, foregut, midgut, hindgut, kidney, heart, and spleen, and a lower level still in the other tissues (*p* < 0.05) (Figure 4).

### 2.4. Quantitative Analyses of Pf_C8α, Pf_C8β and Pf_C9 mRNA Expression Levels during Early Developmental Stages

**Figure 5:**
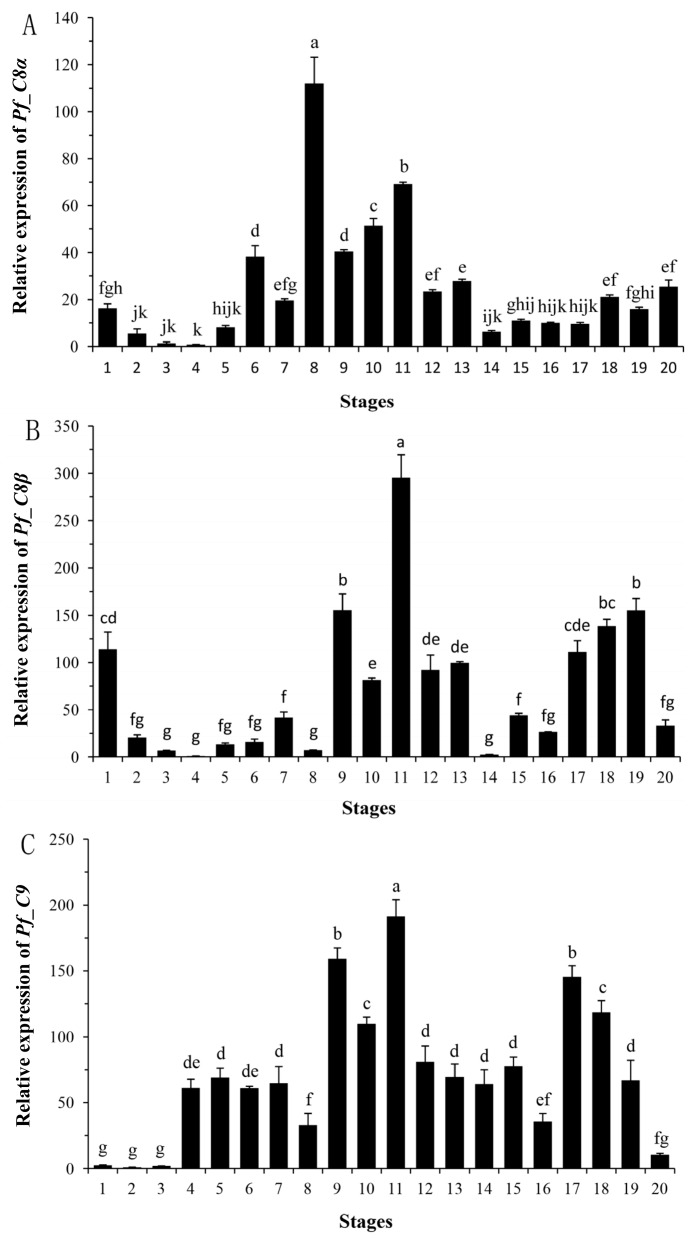
The expression profiles of *Pf_C8α* (**A**); *Pf_C8β* (**B**) and *Pf_C9* (**C**) genes during the embryonic and early larval developmental stages. Abscissa (x-axis) values 1–20 are qPCR products from various developmental stages with (**1**) fertilized egg; (**2**) cleavage; (**3**) blastula; (**4**) gastrula; (**5**) neurula; (**6**) somite appearance; (**7**) muscular effect; (**8**) heart beat; (**9**) blood circulation; (**10**) prophase of hatching; (**11**) newly hatched larval; (**12**) 1 dph; (**13**) 3 dph; (**14**) 5 dph; (**15**) 7 dph; (**16**) 11 dph; (**17**) 15 dph; (**18**) 20 dph; (**19**) 25 dph; (**20**) 30 dph. Different letters above the bars indicate significant difference (Duncan test, *p* < 0.05). dph: days post-hatching.

The relative expression levels of *Pf_C8α*, *Pf_C8β* and *Pf_C9* mRNA were detected during the early developmental stages from fertilized eggs to 30 days post-hatching (dph) larvae (Figure 5). The expression of *Pf_C8α* mRNA was low until the neurula stage (Figure 5A, no. 5), and then increased to a moderate level at the somite appearance stage (Figure 5A, no. 6). It increased significantly to the highest value at the heart beat stage (Figure 5A, no. 8), then significantly decreased to a moderate level at the blood circulation stage (Figure 5A, no. 9) and was maintained in the newly hatched larval stage (Figure 5A, no. 11). The expression of *Pf_C8α* mRNA decreased significantly at 1–3 dph (Figure 5A, nos. 12, 13), and further decreased to a low level at 5 dph (Figure 5A, no. 14), and then fluctuated until 30 dph (Figure 5A, no. 20).

The gene expression of *Pf_C8β* was moderate at the fertilized egg stage (Figure 5B, no. 1). It then significantly decreased to a low level at the cleavage stage (Figure 5B, no. 2) and was maintained until the heart beat stage (Figure 5B, no. 8). It increased significantly to a moderate level at the blood circulation stage (Figure 5B, no. 9), and further increased significantly to its highest level at the newly hatched larval stage (Figure 5B, no. 11). After hatching, the expression of *Pf_C8β* mRNA decreased significantly to a moderate level at 1–3 dph (Figure 5B, nos. 12, 13), further decreased to a low level at 5 dph (Figure 5B, no. 14) and was maintained until 11 dph (Figure 5B, no. 16). Subsequently, the mRNA expression increased significantly to a moderate level at 15 dph (Figure 5B, no. 17), was maintained until 25 dph (Figure 5B, no. 19), and finally decreased to a low level at 30 dph (Figure 5B, no. 20).

The gene expression of *Pf_C9* was low during the early embryonic period from the fertilized egg to the blastula stage (Figure 5C, nos. 1–3). It then increased to a moderate level at the gastrula stage (Figure 5C, no. 4), and was maintained until the muscular effect stage (Figure 5C, no. 7). After a significant decrease at the heart beat stage (Figure 5C, no. 8), the mRNA expression increased significantly to a high level at the blood circulation stage (Figure 5C, no. 9), and reached its highest value at the newly hatched larval stage (Figure 5C, no. 11). After hatching, the expression of *Pf_C9* mRNA decreased significantly to a moderate level at 1–7 dph (Figure 5C, nos. 12–15), and further decreased to a low level at 11 dph (Figure 5C, no. 16). Subsequently, the mRNA expression increased significantly to a high level at 15 dph (Figure 5C, no. 17), and then decreased gradually to a low level at 30 dph (Figure 5C, no. 20).

### 2.5. Quantitative Analysis of Pf_C8α, Pf_C8β and Pf_C9 mRNA Expression Levels in Five Tissues after Challenges of A. hydrophila

**Figure 6:**
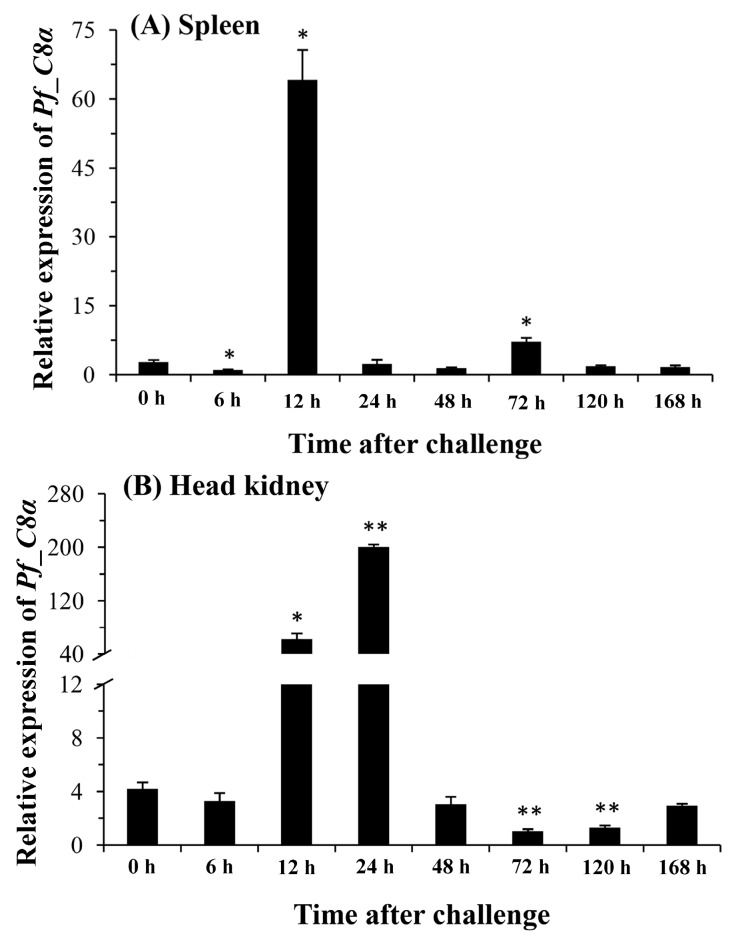

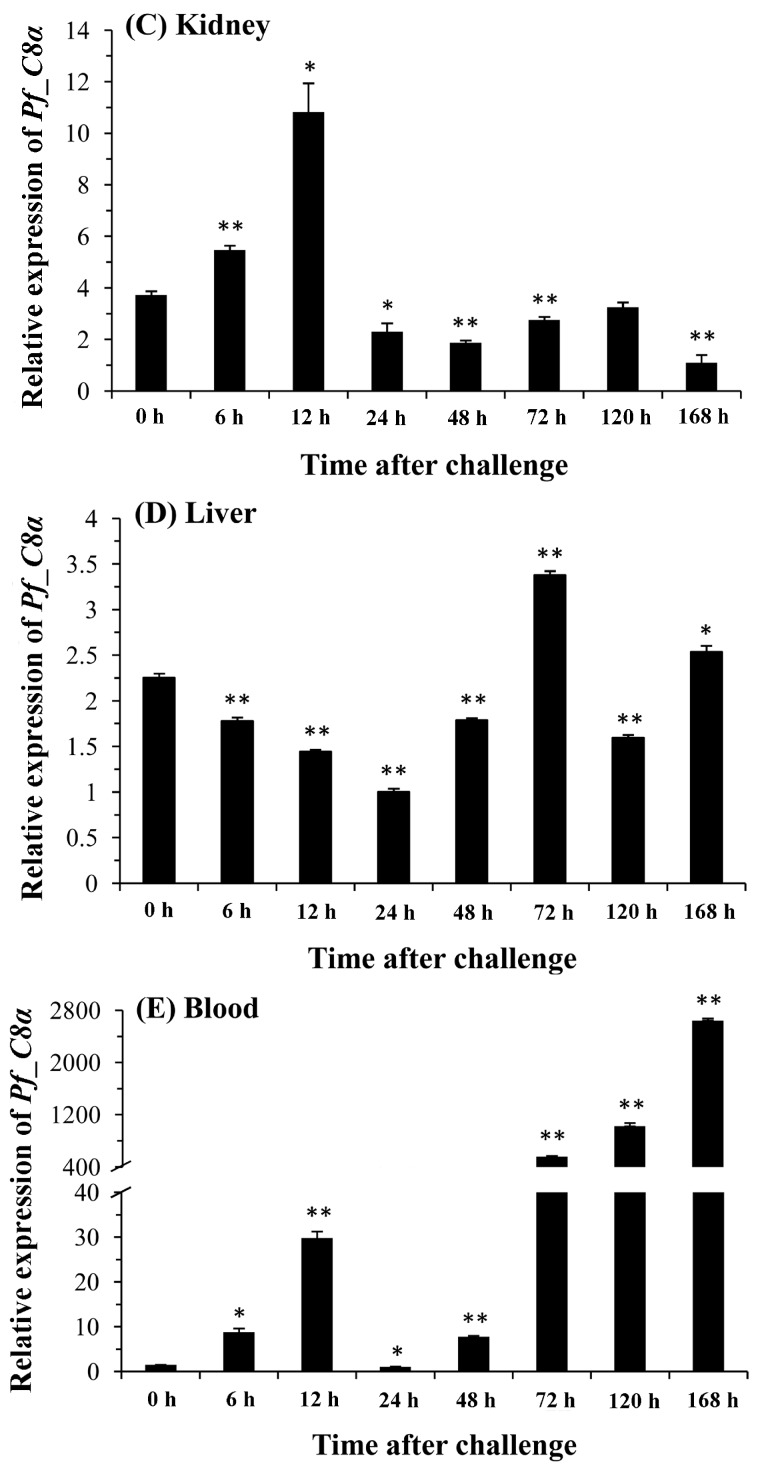
Changes of relative mRNA expression levels of the *Pf_C8α* gene in the spleen (**A**); head kidney (**B**); kidney (**C**); liver (**D**) and blood (**E**) after the *A. hydrophila* challenge. Transcriptional fold changes of *Pf_C8α* at different time points (6, 12, 24, 48, 72, 120 and 168 h) were calculated compared to the control (0 h). Columns and bars represent the means and standard errors, respectively. Significant difference is indicated by asterisks (*: *p* < 0.05, **: *p* < 0.01).

Expression levels of the *Pf_C8α* gene in the spleen, head kidney, kidney, liver, and blood after the *A. hydrophila* challenge were measured by qPCR (Figure 6). In the spleen and kidney, expressions increased significantly to a peak level at 12 h post-injection. This peak appeared at 72 h post-injection in the liver. In the head kidney, the expression level was significantly up-regulated at 12 h and reached a peak at 24 h post-injection. The expression level in the blood increased significantly from 72 to 168 h and reached a peak at 168 h post-injection.

**Figure 7:**
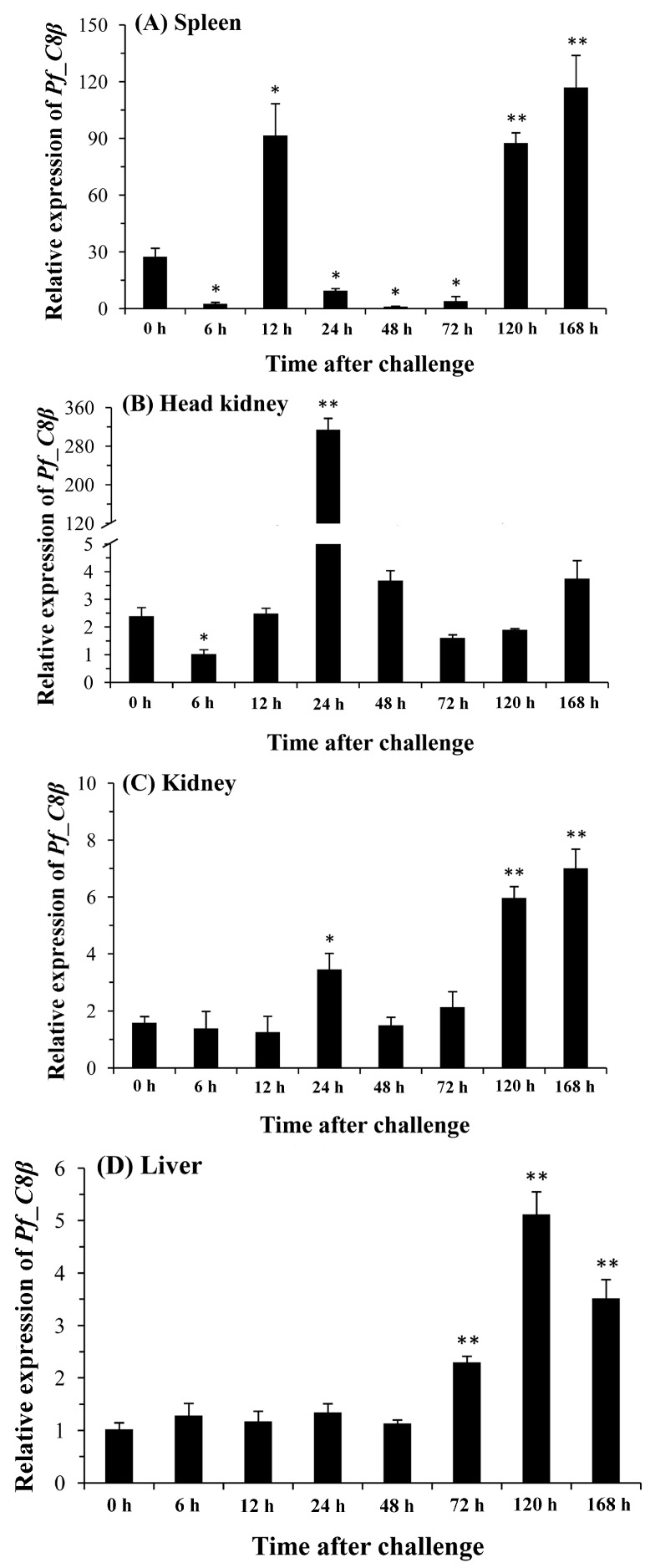

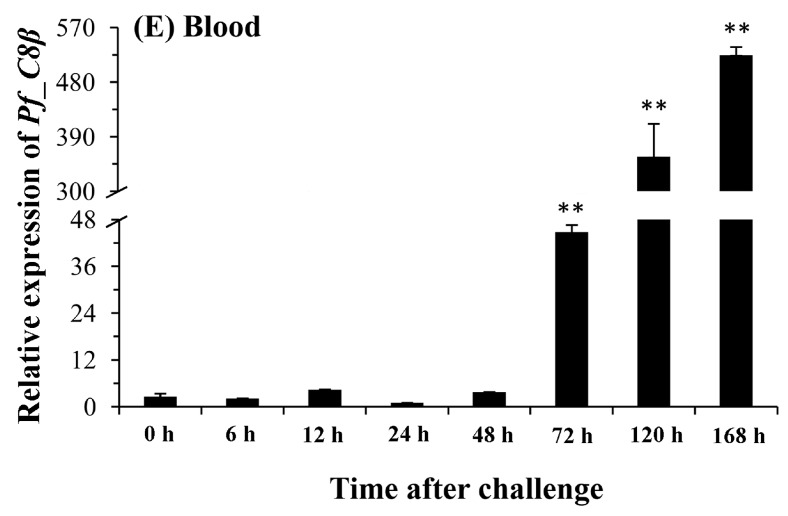
Changes of relative mRNA expression levels of the *Pf_C8β* gene in the spleen (**A**); head kidney (**B**); kidney (**C**); liver (**D**) and blood (**E**) after the *A. hydrophila* challenge. Transcriptional fold changes of *Pf_C8β* at different time points (6, 12, 24, 48, 72, 120 and 168 h) were calculated compared to the control (0 h). Columns and bars represent the means and standard errors, respectively. Significant difference is indicated by asterisks (*: *p* < 0.05, **: *p* < 0.01).

Expression levels of the *Pf_C8β* gene in the spleen, head kidney, kidney, liver, and blood after the *A. hydrophila* challenge were measured by qPCR (Figure 7). In the spleen, kidney and blood, expressions were significantly up-regulated at 120 h and reached a peak level at 168 h post-injection. The expression in the head kidney increased significantly to a peak level at 24 h post-injection. This peak appeared at 120 h post-injection in the liver.

**Figure 8:**
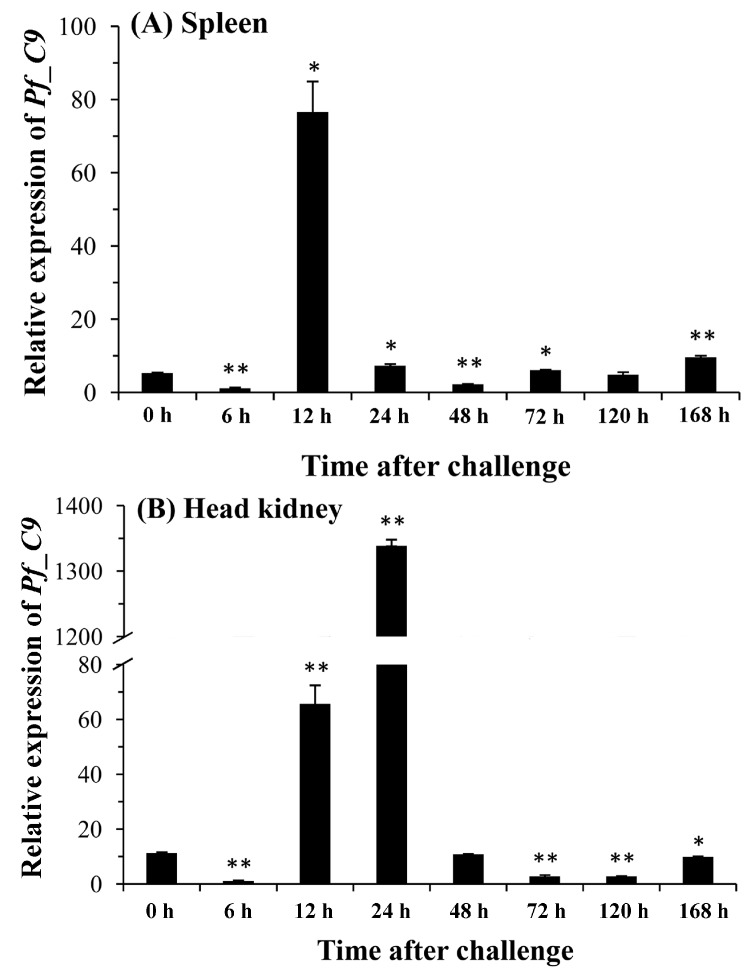

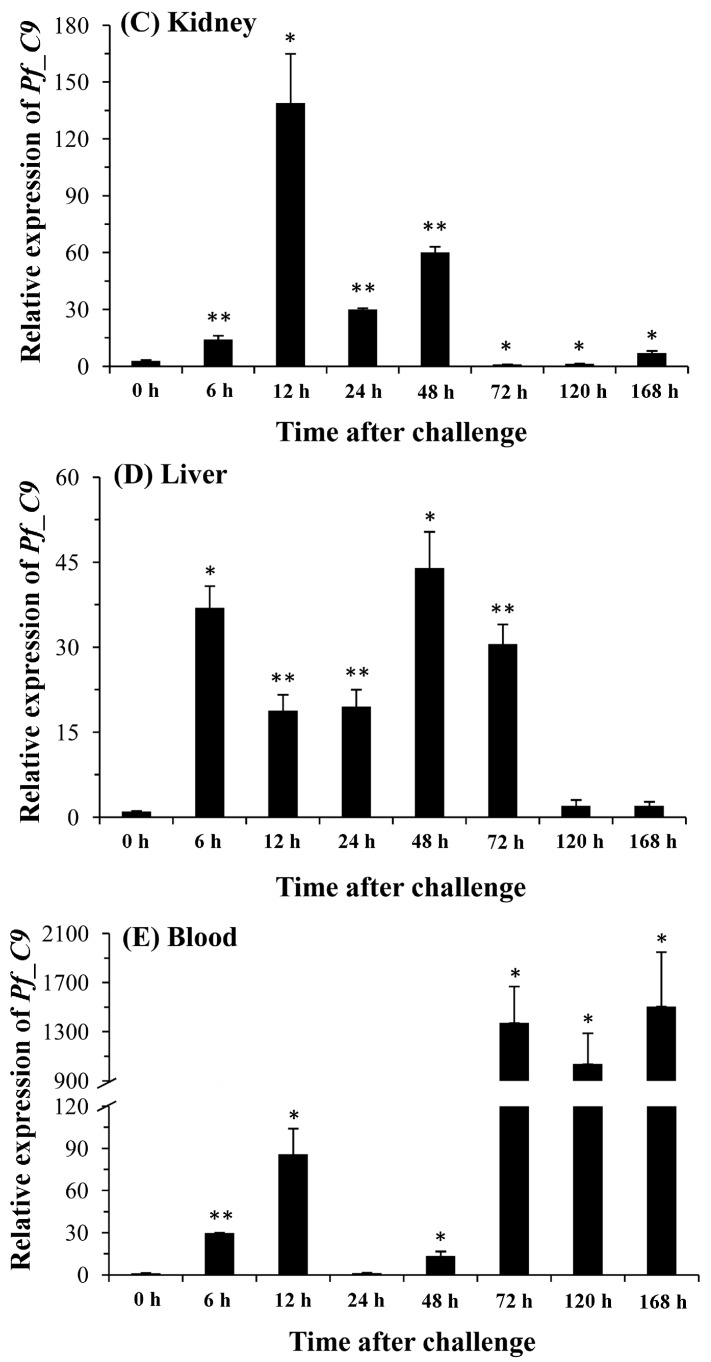
Changes of relative mRNA expression levels of the *Pf_C9* gene in the spleen (**A**); head kidney (**B**); kidney (**C**); liver (**D**) and blood (**E**) after the *A. hydrophila* challenge. Transcriptional fold changes of *Pf_C9* at different time points (6, 12, 24, 48, 72, 120 and 168 h) were calculated compared to the control (0 h). Columns and bars represent the means and standard errors, respectively. Significant difference is indicated by asterisks (*: *p* < 0.05, **: *p* < 0.01).

Expression levels of the *Pf_C9* gene in the spleen, head kidney, kidney, liver, and blood after the *A. hydrophila* challenge were measured by qPCR (Figure 8). In the spleen and kidney, expressions increased significantly to a peak at 12 h post-injection. This peak appeared at 48 h post-injection in the liver. The expression level in the head kidney was significantly up-regulated at 12 h and reached a peak at 24 h post-injection, whereas the expression level in the blood increased significantly from 72 to 168 h and reached a peak at 168 h post-injection.

## 3. Discussion

Yellow catfish is one of the most important economic freshwater species in China [45]. In recent years, the artificial culture of this species has been affected by several kinds of diseases, especially bacterial infection. Because it is known that fish *C8* and *C9* genes are essential components in forming the pore-like MAC of complement on bacterial cells [46]. We isolated, sequenced and characterized three genes of the terminal complement components from yellow catfish to better understand their roles in the innate immune system. The deduced amino acid sequences of the *Pf_C8α*, *Pf_C8β* and *Pf_C9* genes showed greatest similarity to those of other vertebrates. Like most other teleosts, the *Pf_C8α*, *Pf_C8β* and *Pf_C9* genes of yellow catfish all have two TSP 1 domains, an LDLR-A domain, a MACPF domain and an EGF domain. The results suggest that the functionally mature regions of *Pf_*C8α, *Pf_*C8β and *Pf_*C9 are more conserved than their signal peptides, and demonstrate that the three genes are indeed *C8α*, *C8β* and *C9* genes [8,12]. In the C-terminal region, the *Pf*_*C9* gene has a second TSP 1 domain compared with mammalian counterparts, as previously reported for grass carp [27], Japanese flounder [47] and rainbow trout [30]. The second TSP 1 of C9 sequence is one of the distinctive features present in fish [24]. For other TCC genes, the second TSP 1 domain in the C-terminal region is commonly reported, *i.e.*, *C6*, *C7*, *C8* [31,32,33]. Thus, both the *Pf_C8α* and *Pf_C8β* genes have the repeat TSP 1 region in the C-terminal. Moreover, the second TSP 1 of each of the *Pf_*C8α, *Pf_*C8β and *Pf_*C9 sequences contains a C-mannosylation motif (WXXWXXW). The result of phylogenetic analysis of the three complement genes was similar to the BLAST result, and the evolutionary relationship of yellow catfish with other fish and vertebrates in the NJ phylogenetic tree was consistent with the traditional systematics. These unique characteristics and the high homology to other known vertebrates suggest that the *Pf_*C8α, *Pf_*C8β and *Pf_*C9 sequences belong to the MACPF super family of the complement system.

In mammals, the liver tissue is the major organ of synthesis for the majority of the complement components [48]. In rock bream, the expressions of the *C8α*, *C8β* and *C9* genes in the liver were greater compared to other tissues (blood, skin, muscle, heart, brain, head kidney, spleen, kidney, gill, and intestine) [24,26]. In whitespotted bamboo shark (*Chiloscyllium* *plagiosum*), the *C8α* and *C9* genes were extremely high expressed in the liver, with much less or even undetectable expression in other tissues (spleen, heart, fin, gastro, brain, intestine, and muscle) [49,50]. In the current study, *Pf_C8α*, *Pf_C8β* and *Pf_C9* transcripts were most highly expressed in the liver, while their expressions were lower or much lower in the other tissues. Our results support previous findings for other healthy fish: TCC genes are mainly expressed in the liver and constitutively expressed in other tissues, because the liver plays a crucial role in controlling innate immunity, which is highly associated with the immune defense against bacterial infection [51]. Therefore, the liver may be the main source of *Pf*_C8α, *Pf*_C8β and *Pf*_C9 for yellow catfish, while other tissues may also contribute to their synthesis.

There have been limited studies on expressions of *C8α*, *C8β* and *C9* mRNA during the early developmental stages of fish. The complement components are present early in the development of fish, before or soon after hatching [52]. In common carp (*Cyprinus* *carpio* *haematopterus*), expressions of the *C8α* and *C8β* genes were detected at different early developmental stages, whereas expression of the *C9* gene was not detected at the blastula stage, gastrula stage or tail-bud stage, but it was detected at the stage of heart beating and then fluctuated until 5 dph [53]. For other TCC genes, the transcript of *C6* gene in grass carp was detected in unfertilized eggs, the expression level was lower before heart beating and was significantly up-regulated from 1 to 7 dph [35]. Previous research has shown that most of the main immune genes are maternally transferred into eggs during vitellogenesis of fish [54]. Wang *et al.* (2008) found that transcripts of both *C3* and *Bf* genes of the complement system were present in the newly fertilized eggs of zebrafish, and suggested that they are maternal complement components [55]. Lechniak *et al.* (2008) demonstrated that major genome activation in zygotes occurs at the 8–16 cell stage in vertebrates [56]. In the present study, transcripts of the *Pf_C8α*, *Pf_C8β* and *Pf_C9* genes were detected in the newly fertilized egg of yellow catfish, and then they were significantly decreased to a low level at the cleavage stage and were maintained until the blastula stage. These results suggest that the transcripts of these three genes are likely to be derived from maternal copies. In general, newly fertilized eggs are immediately exposed to pathogenic microbes in the aquatic environment. One previous study showed that the complement-mediated killing of pathogenic microbes generally occurs in the early embryonic developmental stages of fishes [55]. Therefore, transferred maternal complement components of yellow catfish might be involved in the early defense against microbial attacks in the aquatic environment during early embryonic development. At the heart beat stage, the expression of the *Pf_C8α* gene was quickly up-regulated to its maximum level and maintained at a relatively high level until the stage of newly hatched larvae. For the *Pf_C8β* and *Pf_C9* genes, expressions were at a relatively high level from the blood circulation stage to the newly hatched larval stage, and expressions were secondly up-regulated at 15–25 dph after a trough at 1–11 dph. These results imply that the *Pf_C8α*, *Pf_C8β* and *Pf_C9* genes might play important roles in the early development of embryos and fry.

TCC genes are immune-related genes which play a crucial role in the innate resistance to bacterial pathogens [46]. A previous qPCR study in rock bream showed that Gram-negative bacteria (*Edwardsiella* *tarda*) can effectively induce expressions of the *C8α* and *C8β* genes in the head kidney and liver. The transcription levels of both *C8α* and *C8β* genes were highest in the liver at 12 h post-injection, and were highest in the head kidney at 24 h post-injection, compared to the un-injected control [26]. After the *Vibrio alginolyticus* challenge in orange-spotted grouper (*Epinephelus* *coioides*), the expressions of *C8β* transcripts in the liver and kidney were, respectively, induced to a peak level at 48 and 12 h post-injection [15]. In grass carp, a qPCR study indicated that *C9* gene expression was effectively triggered by Gram-negative bacteria (*Flavobacterium* *columnare*) in hepatopancreas at 1 day post-injection, whereas the expressions of *C9* in the head kidney and spleen were highest at 7 days post-injection [27]. In rock bream, the transcription level of *C9* in the liver was highest at 12 h post challenge with *E. tarda* [24]. These results indicate that the expressions of a TCC gene in different tissues of a fish species may reach a peak level at different time points after bacterial challenge, as do the expressions of a TCC gene in the same tissue of different species. In this study, the expressions of *Pf_C8α* and *Pf_C8β* transcripts in the liver were respectively induced to a peak level until 72 and 120 h post-injection with *A. hydrophila* compared to the un-injection control, whereas the expression of the *Pf_C9* transcript in the liver was effectively triggered at 6 h post-injection and reached the greatest level at the 48 h post-injection with an approximately 30-fold-high introduction. It took a longer induction time for the *Pf_C8α* and *Pf_C8β* transcriptions in the liver of yellow catfish to reach a peak level than in the liver of rock bream and orange-spotted grouper [15,26]. According to the bacterial challenge results, the expressions of *Pf_C8α*, *Pf_C8β* and *Pf_C9* transcripts were effectively induced in the head kidney of yellow catfish and the transcription levels were highest at 24 h post-injection with very high up-regulation, which is consistent with the bacterial challenge results in the rock bream [26]. In addition, after the *A. hydrophila* challenge in yellow catfish, the expressions of *Pf_C8α*, *Pf_C8β* and *Pf_C9* transcripts showed clear patterns of induction and very high up-regulations at the corresponding time points in the kidney, spleen and blood, demonstrating that they expressed in other immune tissues. These results suggest that the *Pf_C8α*, *Pf_C8β* and *Pf_C9* genes are sensitive to the Gram-negative bacterial challenge and have potential roles in the immune defense system of yellow catfish, of which the liver, head kidney, kidney, spleen and blood tissues may make a contribution to the early defense against invading pathogenic microbes. However, the mechanism whereby bacterial invasion stimulated the complement gene expressions and the relation of interaction between these three genes and other complement genes require further study. The results obtained in the present study help to expand the understanding of expression characteristics and potential functions in innate immune system of teleost TCC genes. In addition, this study has provided basic data for further selective breeding of disease-resistant strain of yellow catfish.

## 4. Materials and Methods

### 4.1. Sampling, Bacterial Preparation and Bacterial Challenge

Fifty adult yellow catfish (one year old) were collected from a commercial fish pond of the Fish Culture Base of the Yangtze River Fisheries Research Institute, Jingzhou city, Hubei Province, China. They were transported to the Fish Breeding Base of Huazhong Agricultural University (HZAU). The fish were fed a commercial diet (Hubei Haid Feeds Company, Wuhan, China) twice daily (8:00 a.m. and 16:00 p.m.) in two circulating water tanks until gene cloning experiments and artificial propagation. The water temperature was kept at 24 °C.

To clone the cDNAs of the *Pf_C8α*, *Pf_C8β* and *Pf_C9* genes and to examine the mRNA expression profiles of three genes in various tissues of yellow catfish, five adult yellow catfish were anesthetized with 300 mg/L tricaine methanesulfonate (MS-222; TCI, Shanghai, China) prior to dissection. The five adult fish used for tissue expression were a mixture of three males and two females. Blood, liver, spleen, head kidney, kidney, foregut, midgut, hindgut, stomach, gonad, heart, gill, brain, muscle, skin, fin, and eye tissues were rapidly sampled for RNA isolation.

To detect the expressions of *Pf_C8α*, *Pf_C8β* and *Pf_C9* genes during the early developmental stages, newly fertilized eggs of yellow catfish were produced from three pairs of parent fish by artificial propagation at HZAU. Fertilized eggs from different pairs of parents were separately reared in different indoor tanks with fresh water at 20–22 °C throughout the experiment. Egg yolk and artemia were used to feed the larvae after hatching and mouth opening formation. Five individuals at the newly fertilized egg, cleavage, blastula, gastrula, neurula, somite appearance, muscular effect, heart beat, blood circulation, prophase of hatching, and newly hatched larval stages, as well as larvae of 1, 3, 5, 7, 11, 15, 20, 25, and 30 dph, were randomly collected from the offspring of a pair of parents for RNA extraction. Microscopic observations were used to verify all embryonic developmental stages.

To examine the expressions of the *Pf_C8α*, *Pf_C8β* and *Pf_C9* genes after bacterial challenge, 60 juvenile yellow catfish were cultivated from the offspring of a pair of parents in HZAU. All fish were reared in a circulating water tank for two weeks at 24 °C, with sufficient dissolved oxygen. The bacterium (*A. hydrophila*) for immune challenge experiments was obtained from the microbiology laboratory of HZAU. The methods of bacterial cultures were as described in the previous study [57]. The *A. hydrophila* were killed with 0.2% formalin for 36 h at 4 °C. Five juvenile fish were injected with 50 μL of phosphate buffered saline (PBS, pH 7.2) as a control group. The other fish were challenged by an intraperitoneal injection of 50 μL of suspended formalin-treated *A. hydrophila* in PBS with a concentration of 1.5 × 10^7^ CFU/mL as the experimental group. Blood, spleen, liver, head kidney and kidney tissues of five fish were collected from the experimental group for RNA extraction after *A. hydrophila* challenge for 6, 12, 24, 48, 72, 120 and 168 h. The experimental fish were anesthetized with 300 mg/L MS-222 piror to dissection. All the experimental procedures were approved by the Institutional Animal Care and Use Committees (IACUC) of HZAU, Wuhan, China.

### 4.2. Total RNA Extraction and cDNA Synthesis

Total RNA was extracted from isolated tissues, embryos and larvae using TRIzol Reagent (Invitrogen, Carlsbad, CA, USA), according to the manufacturer’s instructions. A Nanodrop ND-2000 spectrophotometer (Thermo Scientific, Waltham, MA, USA) was used to determine the purity quotient and concentration of RNA with absorbance measurements of 260 and 280 nm. 1% agarose gel electrophoresis was used to determine the integrity of total RNA. The cDNA was synthesized using 2 μg of total RNA by employing the Revert Aid™ M-MLV Reverse Transcriptase Kit (Promega Corporation, Madison, WI, USA) following the manufacturer’s protocols. The cDNA products were stored at −20 °C.

### 4.3. Cloning of cDNA Sequences for Three Genes

In order to obtain the central sequences for the *Pf_C8α*, *Pf_C8β* and *Pf_C9* genes, gene-specific primer pairs (C8α-F/C8α-R, C8β-F1/C8β-R1 and C8β-F2/C8β-R2, and C9-F/C9-R) were designed according to the result of transcriptome analysis of yellow catfish (Table 2). The PCR reactions consisted of 32 ng cDNA, 0.5 μM of each primer and 50 U Premix *Taq* DNA polymerase (TaKaRa, Dalian, China) in a total volume of 20 μL. The PCR thermal cycling conditions were set as follows: 95 °C for 3 min; followed by 35 cycles at 94 °C for 30 s, optimum temperature (Table 2) for 30 s and 72 °C for 1 min; 72 °C for 10 min.

3′RACE was used to amplify the partial sequences of the *Pf_C8α*, *Pf_C8β* and *Pf_C9* genes. Six gene-specific primers for 3′RACE were designed according to the obtained sequences of the *Pf_C8α*, *Pf_C8β* and *Pf_C9* genes (Table 2).

All primers were synthesized by the Tsingke Biotech Company (Wuhan, China). 1.5% agarose gel electrophoresis was used to check PCR products. The purified PCR products were obtained using an AxyPrep™ gel extraction kit (Axygen, Union City, CA, USA). They were ligated into a pMD18-T vector (TaKaRa, Dalian, China). Subsequently, 10 μL of ligated products were transformed into 100-μL *Escherichia coli* competent cells for 30 min at 16 °C, and they were then mixed with a 890-μL LB (Lysogeny Broth) liquid culture medium. Three selected colonies checked by PCR were sequenced by the Tsingke Biotech Company.

### 4.4. Sequence Analysis

Homologous sequences and the amino acid sequence of *Pf_C8α*, *Pf_C8β* and *Pf_C9* genes were searched for and predicted in GenBank with the BLAST program (Available online: http://www.ncbi.nlm.nih.gov/blast). The putative amino acid sequences were analyzed for the presence of signal peptides using Simple Modular Architecture Research Tool (SMART) (Available online: http://smart.embl-heidelberg.de/). ClustalW (Available online: http://www.genome.jp/tools/clustalw/) and BoxShade (Available online: http://www.ch.embnet.org/software/BOX_form.html) were used for multiple sequence alignments. A neighbor-joining (NJ) phylogenetic tree was constructed based on the putative amino acid sequences using MEGA 5.03 (MEGA Software Development Team, Phenix, AZ, USA).

### 4.5. qPCR and Statistical Analyses

qPCR was used to investigate the temporal and spatial expression analyses of *Pf_C8α*, *Pf_C8β* and *Pf_C9* mRNA. The qPCRs of *Pf_C8α*, *Pf_C8β* and *Pf_C9* were performed using a Roche LightCycler 480 (Roche, Mannheim, Germany). The gene-specific primer pairs for qPCRs were designed based on the amplified sequences of *Pf_C8α*, *Pf_C8β* and *Pf_C9* genes (Table 2). The PCR reaction mixtures of *Pf_C8α*, *Pf_C8β* and *Pf_C9* consisted of a 32-ng cDNA template, 10 μL LightCycler^®^ 480 SYBR Green I Master (Roche, Mannheim, Germany) and 1.0 μM of either gene-specific primer in a total volume of 20 μL. The qPCRs of *Pf_C8α*, *Pf_C8β* and *Pf_C9* were performed in triplicate as: 95 °C for 5 min, followed by 45 cycles at 95 °C for 10 s, optimum annealing temperature (Table 3) for 10 s, 72 °C for 15 s respectively. At the end of each qPCR amplification reaction, melting curve analysis was performed to verify that each detected qPCR product was single. The amplification reaction without the cDNA template was used as a blank control.

The relative expression levels of these three genes were calculated using the comparative *C*_t_ method with the formula 2^−ΔΔ*C*t^ [58]. All data of qPCRs were expressed as the mean ± standard error (SE). One-way analysis of variance (one-way ANOVA) and Duncan’s *post-hoc* test were used to examine the differences among mean expression levels in different tissues and at early developmental stages using SPSS 17.0 software (IBM, Armonk, NY, USA). A *t*-test was performed to examine the differences in expression levels between the control and the experimental group after bacterial challenge. *p*-values less than 0.05 were determined to be statistically significant.

## 5. Conclusions

We cloned the partial cDNA sequences of the *Pf_C8α*, *Pf_C8β* and *Pf_C9* genes from the yellow catfish. The deduced amino acid sequences of the *Pf_*C8α, *Pf_*C8β and *Pf_*C9 showed similarity with other reported teleosts and vertebrates. Ontogenetic expression analyses by qPCR suggested that *Pf_C8α*, *Pf_C8β* and *Pf_C9* may play important roles in the early developmental stages. The *Pf_C8α*, *Pf_C8β* and *Pf_C9* mRNAs were all constitutively expressed at the highest levels in the liver, followed by a relatively low level in the head kidney, foregut, midgut, hindgut, kidney, heart, and spleen, and a lower level still in the other tissues, demonstrating that the liver is the primary site for *Pf_*C8α, *Pf_*C8β and *Pf_*C9 synthesis. Moreover, the *Pf_C8α*, *Pf_C8β* and *Pf_C9* mRNA expressions were found to be up-regulated in the spleen, head kidney, kidney, liver and blood tissues after challenge with *A. hydrophila*, indicating that the *Pf_C8α*, *Pf_C8β* and *Pf_C9* genes play roles in the immune defense system of yellow catfish. However, the mechanism whereby bacterial invasion stimulated the complement gene expressions and the relation of interaction between these three genes and other complement genes require further study. The findings obtained in this study have provided valuable information to increase our understanding of the yellow catfish immune system, and help to expand the understanding of expression characteristics of teleost TCC genes.

## Figures and Tables

**Table 1:** Different vertebrate species and their GenBank accession numbers of C8α, C8β and C9 sequences used for multiple alignment and phylogenetic analysis in this study.

Protein	Species	GenBank Accession no.
C8α	*Esox lucius*	XP_010885181.1
	*Oncorhynchus mykiss*	NP_001118096.1
	*Oplegnathus fasciatus*	AFZ93888.1
	*Siniperca chuatsi*	AKA66305.1
	*Clupea harengus*	XP_012681433.1
	*Pelteobagrus fulvidraco*	KT588317
	*Danio rerio*	AAH78409.1
	*Xenopus (Silurana) tropicalis*	AAH74554.1
	*Gallus gallus*	XP_426667.2
	*Mus musculus*	AAH27748.1
	*Bos taurus*	AAI12636.1
	*Homo sapiens*	AAI32914.1
	*Oryctolagus cuniculus*	NP_001075724.1
C8β	*Homo sapiens*	NP_000057.2
	*Macaca mulatta*	XP_001114456.1
	*Oryctolagus cuniculus*	P98137.1
	*Rattus norvegicus*	NP_001178688.1
	*Pelteobagrus fulvidraco*	KT588318
	*Danio rerio*	NP_001243652.1
	*Esox lucius*	XP_010885201.1
	*Oncorhynchus mykiss*	AAL16647.1
	*Neolamprologus brichardi*	XP_006786991.1
	*Paralichthys olivaceus*	BAA86877.1
C9	*Oryctolagus cuniculus*	NP_001075815.1
	*Homo sapiens*	AAH20721.1
	*Sus scrofa*	NP_001090917.1
	*Rattus norvegicus*	AAB38023.1
	*Xenopus (Silurana) tropicalis*	AAI59018.1
	*Danio rerio*	XP_005171925.1
	*Ctenopharyngodon idella*	ABN49522.1
	*Pelteobagrus fulvidraco*	KT454382
	*Plecoglossus altivelis*	CBX31962.1
	*Oncorhynchus mykiss*	NP_001117898.1
	*Xiphophorus hellerii*	AEJ08068.1
	*Oplegnathus fasciatus*	AFU81223.1
	*Paralichthys olivaceus*	BAA86878.1
	*Takifugu rubripes*	AAC60288.1

**Table 2:** Primers used for cloning of *C8α*, *C8β* and *C9* cDNA in *Pelteobagrus* *fulvidraco*.

Name of Primer	Sequence of Primes (5′→3′)	*T*_a_ (°C)	Application
C8α-F	ATGAGGCAGTATATCTCTACTTCTGG	60	cDNA cloning of *C8α* gene
C8α-R	TCAGCAGCGTCTTGTCTGCA		
C8α-3′RACE outer	CTGTCAGATGGCGGGTGGCA	58	3′RACE (1st round PCR)
C8α-3′RACE inner	CTTCAGTTCGCCTGCTCCCG	58	3′RACE (2nd round PCR)
C8β-F1	CGCCGTCACGCTACTCAT	60	cDNA Cloning of *C8β* gene
C8β-R1	GTGGTCAAAGGTTCCCCC		
C8β-F2	CTTTCGTTTCGTGCCT	50	cDNA cloning of *C8β* gene
C8β-R2	GTGTATTGCTGGATGTTGTA		
C8β-3′RACE outer	CCTGCGAACTCTCCCACT	55	3′RACE (1st round PCR)
C8β-3′RACE inner	GCTTGGGGGAACCTTTGAC	55	3′RACE (2nd round PCR)
C9-F	ATGAGGATGTTGATGTCAAT	56	cDNA cloning of *C9* gene
C9-R	TTAACAATATTCGTCGCTGA		
C9-3′RACE outer	CACCCCTACTGAGTCATCGG	58	3′RACE (1st round PCR)
C9-3′RACE inner	ATTACAGACCACAGCACCAC	54	3′RACE (2nd round PCR)
Oligo(dT)_17_	GACTCGAGTCGACATCGA(T)_17_		Universal primer for 3′RACE
linker adapter	GACTCGAGTCGACATCG		Universal primer for 3′RACE

*T*_a_, annealing temperature; RACE: rapid amplification of cDNA ends.

**Table 3:** Primers used for qPCRs of *C8α*, *C8β* and *C9* mRNA and *β-actin* mRNA in *Pelteobagrus* *fulvidraco*.

Name of Primer	Sequence of Primes (5′→3′)	*T*_a_ (°C)	Application
C8α-DLF	TGCCTGAGCAATACGACT	58	qPCR of *C8α* mRNA
C8α-DLR	CGAGCACCACTATGTAATCTA		
C8β-DLF	CAAGGAGCCAACAGAAGA	55	qPCR of *C8β* mRNA
C8β-DLR	TGAAGGCAAAGGAGACAG		
C9-DLF	TTCATTGCTGAGCGAACT	62	qPCR of *C9* mRNA
C9-DLR	ATTTGTGGTGCTGTGGTC		
β-actin-F	TCCCTGTATGCCTCTGGTGGT	58	qPCR of *β-actin* mRNA
β-actin-R	AAGCTGTAGCCTCTCTCGGTC		

*T*_a_, annealing temperature; DL: real time PCR.
